# The influence of competitive anxiety of Chinese elite swimmers

**DOI:** 10.3389/fpsyg.2024.1392137

**Published:** 2024-08-13

**Authors:** Yuhang Zhou, Zhenyu Jin, Yuhong Wen

**Affiliations:** ^1^School of Recreational Sports and Tourism, Beijing Sport University, Beijing, China; ^2^Key Laboratory of Sport Training of General Administration of Sport of China, Beijing Sport University, Beijing, China; ^3^China Swimming College, Beijing Sport University, Beijing, China

**Keywords:** competitive anxiety, trait anxiety, elite athletes, swimming, sport

## Abstract

**Background:**

Competitive anxiety is a common stress response in competitive environments, influencing sports performance, particularly among elite swimmers.

**Purpose:**

This study aims to examine how the characteristics of competitive anxiety impact sports performance across different phases of competitive preparation, alongside its correlation with trait anxiety.

**Methods:**

A longitudinal research design, integrating quantitative and qualitative approaches, was employed with 20 swimmers from the Chinese national swimming team participating in both the 2023 Fukuoka World Championships and the Hangzhou Asian Games. The mental readiness form-3 (MRF-3) scale was employed at four time points during the preparation period for longitudinal tracking, complemented by the trait anxiety inventory (T-AI) scale to assess trait anxiety.

**Results:**

The study identified a negative correlation between cognitive anxiety and somatic anxiety among elite swimmers, with confidence demonstrating a positive correlation. Additionally, there was a significant between trait anxiety and competitive anxiety (*p* < 0.05). Variations in competitive anxiety were found at different stages of the preparation cycle (*F* = 15.074; 11.627; 19.552, *p* < 0.05), impacting sport performance.

**Conclusion:**

To optimise performance, tailored psychological intervention programs should be developed and implemented to address the distinct characteristics of competitive anxiety experienced by elite swimmers throughout the preparation phases.

## 1 Introduction

Swimming, a cornerstone of China’s competitive sports strategy, is a crucial event in the Olympic Games and one of the most competitive sports (Chen, 2021). The 14th Five-Year Plan for Sports Development emphasises the need to vigorously promote foundational sports such as track and field and swimming (General Administration of Sport, 2021), with a focus on continually improving competitiveness. Research indicates that enhancing swimmers’ competitiveness involves physical, psychological, and biomechanical factors (Amara et al., 2021a,b; Lopes et al., 2021). Swimming’s prominence in the Olympics has led researchers to investigate the attributes that distinguish elite swimmers from the rest (Barbosa et al., 2010). For athletes, competition tests not only their athletic ability, but also their psychological resilience. At the highest levels, competitive sport often becomes a context of psychological strength. Studies suggest that while technical and physical skills set the performance range of athletes, psychological factors significantly influence their competitive status and performance (Jones et al., 2018). Moreover, numerous studies have shown that competitive anxiety, a common psychological issue, can impact athletes’ performance.

Anxiety is often defined as a negative psychological state experienced when completing a task under perceived pressure (Martens et al., 1990). It is characterised by tension and worry and is associated with physical activation or arousal (Nascimento et al., 2012). Spielberger categorized anxiety into two types: Trait anxiety, which is a relatively stable personality tendency, and state anxiety, which is a temporary, situation-specific condition (Spielberger, 1966).

According to Baumeister (1984), competitive anxiety is an athlete response to a stressful competitive situation. It involves a series of cognitive appraisals, behavioural responses, and physiological arousals to perceived stressors specific to the sport (Martens et al., 1990), which add to the general state anxiety. Competitive anxiety, one of the many emotions athletes may experience during competition, is a key constraint on performance. Initially, competitive anxiety was categorized into cognitive anxiety and somatic anxiety. Cognitive anxiety refers to the subjective worry about a threatening situation occurring immediately before, during, or after the competition, and stems from negative self-evaluations or performance expectations. Somatic anxiety is the emotional experience of the autonomic nervous system, caused directly by its arousal during the same periods. Martens et al. (1990) later added the dimension, self- confidence, which encompasses athletes’ beliefs about their potential of success before, during, and after a competition.

Competitive anxiety affects sport performance. The multidimensional theory of anxiety proposed by Morris et al. (1981) explains this relationship, suggesting that sport performance is related to athletes’ trait and state anxiety. Athletes with high levels of trait anxiety are likely to experience high levels of state anxiety during competition. A meta-analysis, which included 3,589 athletes across 77 sports, found that anxiety generally has a negative correlation with sport performance (Kleine, 1990). These findings align with those of Lee et al.’s (2020), who also noted the detrimental effect of anxiety on performance (Lee et al., 2020). Scholars have specifically suggested that anxiety affects swimming performance (Dalamitros et al., 2019; Clemente-Suárez et al., 2021). This study investigates the relationship between sport performance and competitive anxiety, using World Swimming Federation points to quantify sport performance. These points represent a swimmer’s best mark relative to the world best mark (Morais et al., 2020).

Most current research on the effects of competitive anxiety analyses the links between competitive anxiety, athletes’ demographic characteristics, and sporting situations (Lee et al., 2020). Some studies have found higher levels of competitive anxiety in females than in males (Amaro and Brandão, 2023), as evidenced by higher scores on cognitive and somatic anxiety and lower scores on self-confidence. However, no significant differences have been found between the sexes (Guillén and Álvarez-Malé, 2010). In some cases, men exhibit higher levels of anxiety than women in team sports such as football (Esteves et al., 2021). Sporting situations can significantly impact athletes’ competitive anxiety. For example, the presence or absence of spectators can significantly impact young athletes’ anxiety levels. Athletes with strong competition confidence often thrive on the attention of a large audience, using it as motivation to achieve excellence (Cai, 2015).

In addition to individual differences affecting levels of competitive anxiety, previous research has shown that training also impacts athletes’ psychological states (Köroglu and Yigiter, 2016). As a result, scholars have begun monitoring the effects of training volume on psychological states (Freitas et al., 2014). The findings suggest that training cycles, which include changes in training load, correspond to specific emotional changes (Winsley and Matos, 2011). This indicates that training load directly influences the psychological state of the athlete. In high-level swimmers, anxiety states during the training period before major competition show a clear trajectory, characterised by either high intensity training (Morgan and Hayward, 1988) or sharply reduced training load (O'Connor et al., 1989). This is evidenced by high training loads correlating with highly competitive anxiety values (Chortane et al., 2022).

Some scholars have emphasised through meta-analysis that appropriate intervention for competitive anxiety can prolong athletes’ sports life (Reynoso-Sánchez and Hoyos-Flores, 2023) and improve their career satisfaction (Rice et al., 2019). Excessive competitive anxiety can negatively affect athletes’ training, pre-competition preparation, and performance. Therefore, it is essential to control athletes’ competitive anxiety to optimise their performance (Pulido et al., 2021). Addressing and exploring the competitive anxiety of elite swimmers during critical preparation periods, and implementing effective interventions to improve their mental health, is a crucial issue for the development of competitive sports.

Most existing studies have explored athletes’ competitive anxiety in relation to demographic variables such as gender but have not focused on elite swimmers at different points in time to accurately capture the characteristics of their competitive anxiety levels throughout the preparation cycle (Sanader et al., 2021). This approach is necessary to propose targeted optimisation measures. Therefore, tthe present study aims at analysing the demographic characteristics of elite swimmers’ competitive anxiety during their preparations for the World Championships and the Asian Games, as well as to examine competitive anxiety at different points in time and its relationship with sport performance, aims to examine how the characteristics of competitive anxiety impact sports performance across different phases of competitive preparation, alongside its correlation with trait anxiety.

Based on the relevant literature, we hypothesised:

*H1*: There are differences in the level of competitive anxiety among elite swimmers with different demographic characteristics;

*H2*: Somatic and cognitive anxiety are significantly positively correlated and significantly negatively correlated with self-confidence;

*H3*: Trait anxiety predicts competitive anxiety;

*H4*: There are significant differences in the levels of competitive anxiety among elite swimmers at different time points;

*H5*: Competitive anxiety influences sport performance.

## 2 Materials and methods

### 2.1 Methodological approach

Methodology is “the theory of the method of understanding and transforming the world” (Shao and Chen, 2002). In sport science research positivist methodology, characterised by a trend towards quantification, is increasingly dominant. Shao, Chen, and others emphasised the need to strengthen this quantitative approach to improve the overall level of sports and social sciences. This improvement should start with the accumulation of empirical evidence and then move towards theoretical development (Zhang, 2005). This is why the current paper relies on the questionnaire survey method. However, constructivists argue that facts are not external to our existence but are constructed through our interactions with the outside world and others. They see knowledge as something that is generated rather than discovered. The qualitative research paradigm emphasises that the formation and development of knowledge is not limited by inherent rational principles or purely rational inference (Zhang, 2002). Instead, knowledge is built by the conscious actions of individuals in their everyday interactions, a process of “negotiation.” Given that the elite swimmers in this study are at the Olympic delegation level, there is considerable individual variation. Combining both quantitative and qualitative perspectives allows for a richer and more comprehensive study.

### 2.2 Subjects

The subjects of this study were 20 athletes from the Chinese National Swimming Team (subject information is shown in Table 1).

**Table 1:** Demographic characteristics of all the participants.

Variable	Category	Frequency	Percentage	Percentage with WR
Gender	Male	10	50%	93.18%
	Female	10	50%	93.26%
Age	<19	5	25%	92.64%
	≥19and < 21	7	35%	93.89%
	≥21	8	40%	93%
Training years	<9	7	35%	93.97%
	≥9and < 13	7	35%	91.39%
	≥13	6	30%	94.48%
Sports grade	World-class	14	65%	95.53%
	National	6	35%	87.83%

“Percentage with WR” is refer to swimmers’ World Aquatics Points percentage of the world record. The World Aquatics Points Table allows comparisons of results among different events. The World Aquatics Point Scoring assigns point values to swimming performances, more points for world class performances typically 1,000 or more and fewer points for slower performances.

*A priori* power analysis (G * Power 3.1.9.6, Heinrich Heine Universität Düsseldorf, Düsseldorf, Germany) yielded a sample size of at least 12 swimmers in one time point to detect medium effects, assuming a power of 0.8 and alpha of 0.05. Thus, result of power analysis provided evidence that sample size of the present study is acceptable.

All the World Aquatics points were retrieved from publicly accessible database “swimrankings.net.” The database lists are the results of registered races which are in accordance with the official World Aquatics rules (World Aquatics, 2023), including electronical time keeping and limits to in-pool current (Born et al., 2020). In this study, the 2023 World Aquatics points reference values were used.

The inclusion criteria for this study were: (1) age 15 years or older; (2) qualification for both the Fukuoka World Championships and the Hangzhou Asian Games in a single event; and (3) ability to complete the surveys at all time points in this study. A total of 30 swimmers from the Chinese swimming team met the first two criteria 1 and 2, but 10 were excluded because they could not complete the questionnaires at all required time points, thus not meeting the third criterion.

Elite athletes are those who compete at the professional and Olympic levels (Shi and Ma, 2022). The selection process of the Chinese delegation for the Fukuoka World Championships and Hangzhou Asian Games took place during the National Swimming Championships in May. The high caliber of swimmers selected for the two delegations aligns well with the elite characteristics of the swimmers in this study. These two events, being significant competitions preceding the Olympic Games preparatory cycle, are highly valued by all stakeholders.

All respondents signed the Informed Consent form, which was approved by the Sports Science Experiment Ethics Committee, study name “The influence of competitive anxiety of elite swimmers” (No. 2023261H).

### 2.3 Methods

#### 2.3.1 Trait anxiety scale

The State–Trait Anxiety Inventory (STAI), developed and revised by Charles Spielberger, comprises two subscales: the State Anxiety Inventory (S-AI) and the Trait Anxiety Inventory (T-AI) (Spielberger, 1966). Only the Trait Anxiety Inventory (T-AI) was used in this study, consisting of 20 items. Items, including 11 positively scored items and 9 negatively scored items, rated on a four-point scale. Higher scores indicate higher levels of trait anxiety in individuals. x^2^/df = 3.90, TLI = 0.92, CFI = 0.94, RMSEA = 0.05, SRMR = 0.05, Indicating good construct validity of the scale, the Cronbach a = 0.86. The scale was administered to all participants in a calm state of mind, without considering their training schedules. Validated factor analysis of the scale.

#### 2.3.2 Competitive anxiety scale

The study participants’ state anxiety was assessed using the Mental Readiness Form-3 (MRF-3) (Krane, 1994). The MRF-3 offers a less invasive and time-consuming alternative to longer questionnaires, making it suitable for situations requiring repeated measures and shorter completion times (Wilson et al., 2009; Beseler et al., 2016). It consists of three scales, each a two-ended continuum, ranging from 1 to 11. These scales assess cognitive anxiety (ranged from relaxed to worried), somatic anxiety (ranged from relaxed to tense), and assertiveness (ranged from confident to fearful). All participants completed the MRF-3 pre-test in a quiet setting, establishing the baseline value (T0). A random measure of competitive anxiety during a high-volume training session was recorded as T1. Anxiety levels were measured 30 min before the Fukuoka World Championships (T2); and 30 min before the Hangzhou Asian Games was recorded (T3).

#### 2.3.3 Semi-structured interview

The interview method is commonly used in qualitative research to gather information from participants through questioning (Chen, 2006). For this study, a semi-structured interview approach was chosen. In semi-structured interviews, the interviewer asks questions based on a prepared outline but maintains flexibility in the interview’s structure. The interviewer can adjust the order of questions according to the flow of the conversation and the respondents’ answers. This method allows respondents to actively participate in the interview process and raise their own questions.

Respondents’ selection criteria were as follows: homogeneity, comprehensive information coverage, and informed consent to participate in the survey. The first author, who was a member of the Chinese swimming team’s safeguarding team, had sufficient time and access to the athletes. Consent was obtained from the coaches of each interviewee, with a detailed explanation of the ongoing research and the option to accept or refuse participation at any time. Final consent was obtained from all coaches and interviewees.

Six interviewees participated in the study, with Table 2 displaying the information gathered from the interviews. The demographic characteristics examined included the participants’ ID, gender, age, training years in competitive sports, and career-best performance as swimmers. The interviews took place in an office setting, scheduled at times when all participants in a calm state of mind, without considering their training schedules., lasting between 30 to 60 min.

**Table 2:** Interviewee information.

ID	Gender	Age	Training Years	Best performance
A1	Female	25	19	Olympic Champion
A2	Female	21	15	Olympic Champion
A3	Male	24	19	World Champion
A4	Female	19	12	World Champion
A5	Male	16	11	World Champion
A6	Male	20	13	Asian Games Champion

Before the interviews, we asked the participants to sign an informed consent form, assuring them the data collected would be used only for academic research and that their personal information would be protected. We also asked for their consent to be recorded. Next, we explained the purpose and contents of the interviews. We then proceeded with based on the interview outline, which included topics such as “mental state during the preparation period” and “strategies for coping with competition anxiety.” Finally, we organised the data collected from the interviews.

### 2.4 Statistical analysis

visual inspection of histogram was used to test the normality of the sample. We used percentages, means, and standard deviations to analyse demographic information, trait anxiety, state anxiety characteristics, and data distribution of the samples. Independent t-tests were conducted to explore the variability of trait anxiety and state anxiety across different demographics and the differences in competitive anxiety across various sport performances. One-way analysis of variance (ANOVA) was used to explore the variability of state anxiety under different time points. Pearson’s correlation tests were used to investigate the correlations between trait anxiety and state anxiety in different contexts as well as the correlations between various dimensions of state anxiety during periods of high training volume. Setting *p* < 0.05 as statistically significant. Using the statistical package Statistical Package for the Social Sciences (SPSS) - IBM 22.0 (SPSS Inc., Chicago, IL, United States). Datas are presented using tables.

## 3 Results

Table 3 showed that no significant difference between male and female swimmers in trait anxiety and state anxiety dimensions (*p* > 0.05). However, on average, male swimmers had higher levels of both trait anxiety and state anxiety compared to female swimmers, but also exhibited higher levels of self-confidence.

**Table 3:** Gender differences in trait anxiety and competitive anxiety (*N* = 20).

	Gender (M ± SD)		*t*	*p*
	Male	Female		
Trait anxiety	46.40 ± 10.69	42.00 ± 6.02	1.134	0.272
Cognitive anxiety	6.85 ± 1.81	6.50 ± 1.92	0.840	0.404
Somatic anxiety	7.03 ± 2.06	6.83 ± 1.74	0.470	0.640
Confidence	5.45 ± 1.95	6.13 ± 2.08	−1.499	0.138

Previous research has emphasised the importance of investigating the impact of competitive anxiety levels on sports performance. According to Ruiz-Navarro et al. (2023), this study classifies participants into two groups based on their World Aquatics Points at the Fukuoka World Championships and the Hangzhou Asian Games to distinguish between successful and unsuccessful performances. The classification criteria are as follows:

i) The first level is based on the A qualifying standards set to participate at the international events, which correspond to ≥875 World Aquatics Points. ii) The second level is based on the B qualifying standards set to participate at the international events, which correspond to 794 World Aquatics Points.

There was no significant difference in competitive anxiety between swimmers at the Fukuoka World Championships and the Hangzhou Asian Games based on sports performance (World Aquatics points). However, on average, athletes who performed well at the Fukuoka World Championships had higher cognitive anxiety but lower somatic anxiety. In contrast, athletes who excelled at the Asian Games exhibited lower levels of state anxiety (Tables 4, 5).

**Table 4:** The difference of competitive anxiety under sports performance in T2 (*N* = 20).

	World aquatics points (M±SD)		*t*	*p*
	Level 1 (*n*=11)	Level 2 (*n*=9)		
Cognitive anxiety	7.36 ± 1.80	7.22 ± 0.97	0.211	0.835
Somatic anxiety	7.55 ± 1.92	8.11 ± 1.27	−0.758	0.458
Confidence	8.36 ± 2.42	7.89 ± 0.93	0.554	0.586
Trait anxiety	45.00 ± 6.84	43.22 ± 11.00	0.443	0.663

T2 is anxiety levels were measured 30 minutes before the Fukuoka World Championships.

**Table 5:** The difference of competitive anxiety under sports performance in T3 (*N* = 20).

	World aquatics points (M ± SD)		*t*	*p*
	Level 1 (*n*=14)	Level 2 (*n*=6)		
Cognitive anxiety	6.64 ± 1.78	7.00 ± 0.89	−0.462	0.650
Somatic anxiety	6.93 ± 1.90	7.67 ± 1.03	−0.888	0.386
Confidence	7.43 ± 2.21	7.17 ± 0.75	0.280	0.783
Trait anxiety	45.43 ± 9.41	41.33 ± 6.77	0.958	0.351

T3 is anxiety levels were measured 30 minutes before the Hangzhou Asian Games.

Table 6 shows that significant differences were found in all dimensions of competitive anxiety at different time points (*p* < 0.01). Competitive anxiety was highest during the World Championships, followed by the Asian Games period and the high training volume period, all of which were higher than the baseline values.

**Table 6:** The difference of state anxiety at different points in time (*N* = 20).

	Different points in time (M ± SD)				*F*	*p*
	T0	T1	T2	T3		
Cognitive anxiety	5.00 ± 1.75	6.55 ± 1.00	8.15 ± 1.39	7.00 ± 1.75	15.074	<0.001**
Somatic anxiety	5.25 ± 2.17	7.45 ± 1.61	8.10 ± 1.21	6.90 ± 1.21	11.637	<0.001**
Confidence	6.75 ± 1.62	4.85 ± 1.46	4.15 ± 1.27	7.40 ± 1.82	19.552	<0.001**

All participants completed the MRF-3 pre-test in a quiet setting, establishing the baseline value (T0). A random measure of competitive anxiety during a high-volume training session was recorded as T1. Anxiety levels were measured 30 minutes before the Fukuoka World Championships (T2); and 30 minutes before the Hangzhou Asian Games was recorded (T3); * *p*<0.05; ** *p*<0.001.

**Figure 1:**
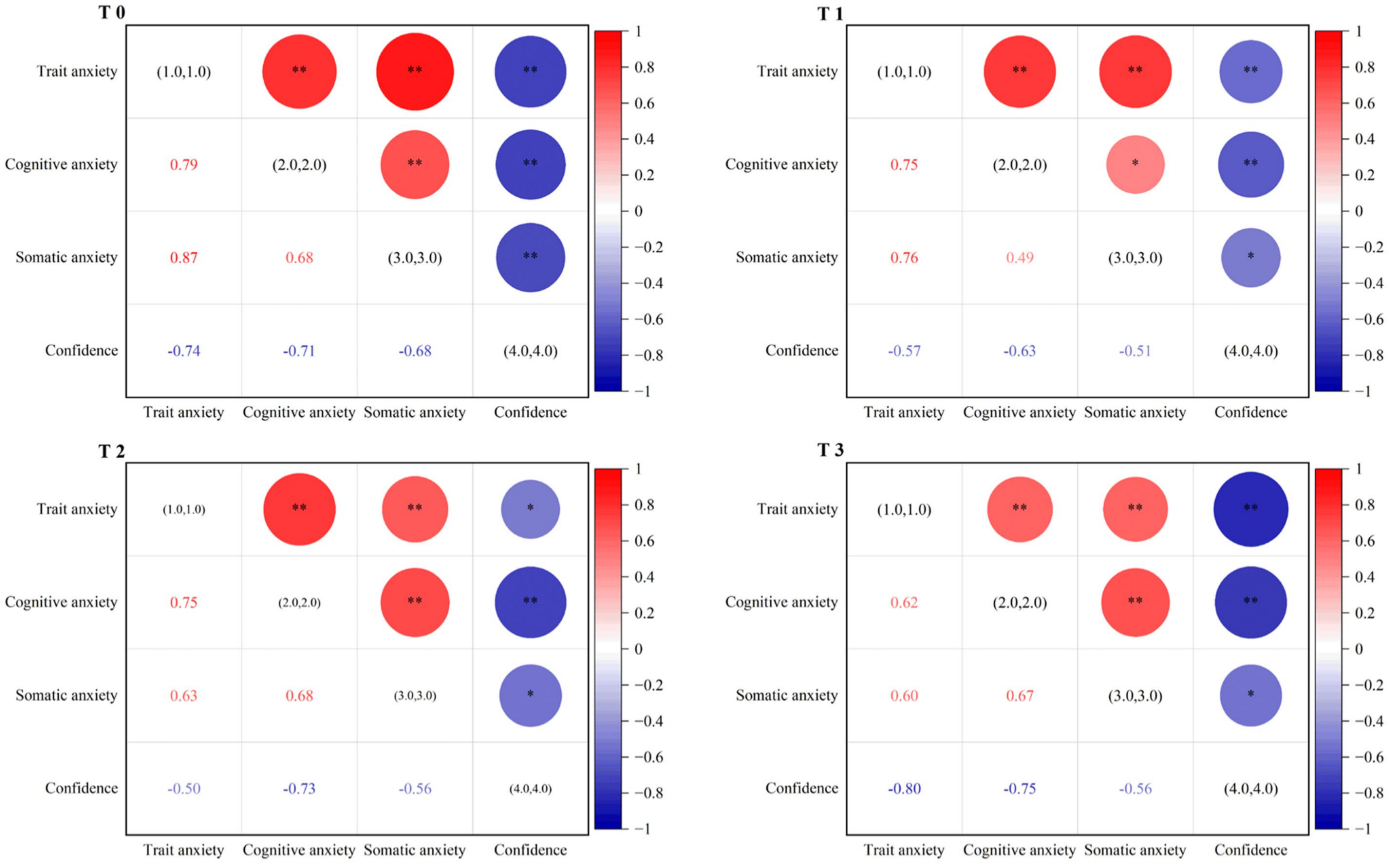
Correlation of competitive anxiety and trait anxiety in T0, T1, T2, and T3. All participants completed the MRF-3 pre-test in a quiet setting, establishing the baseline value (T0). A random measure of competitive anxiety during a high-volume training session was recorded as T1. Anxiety levels were measured 30 minutes before the Fukuoka World Championships (T2); and 30 minutes before the Hangzhou Asian Games was recorded (T3); *p < 0.05; **p < 0.01.

Figure 1 display the relationships between trait anxiety and state anxiety at different time points, the r coefficient and significance are shown in the figure. The findings indicate that cognitive anxiety and somatic anxiety are positively correlated with trait anxiety, while self-confidence is negatively correlated with trait anxiety (*p* < 0.05).

Further analysis of the correlations among the dimensions of competitive anxiety revealed that cognitive anxiety was positively correlated with somatic anxiety and negatively correlated with self-confidence (*p* < 0.05).

Based on the correlation between trait anxiety and competitive anxiety, covariates were added to the regression analysis. The results showed that training years positively correlated with cognitive anxiety during the World Championships; training years positively correlated with somatic anxiety during the Asian Games (Tables 7, 8).

**Table 7:** Linear regression analysis during world championships.

	Unstandardized coefficients		Standardized coefficients	*t*	*p*	Collinearity diagnostics	
	*B*	*SE*	*Beta*			VIF	Tolerance
Constant	0.442	1.361	–	0.325	0.750	–	–
Gender	0.013	0.396	0.005	0.033	0.974	1.260	0.794
Age	−0.068	0.236	−0.040	−0.288	0.777	1.123	0.891
Training years	0.768	0.261	0.457	2.940	0.010*	1.425	0.702
Trait anxiety	0.143	0.022	0.903	6.471	<0.001**	1.147	0.872
*R* ^2^	0.745						
*Adjust R*^2^	0.677						
*F*	*F* (4,15) = 10.962, *p* = 0.000						
D-W	1.448						

Dependent variable: cognitive anxiety.

VIF is designed to determine collinearity problems, and a value less than 5 indicates no collinearity. Tolerance = 1/VIF and the value less than 0.2 indicates collinearity; * *p*<0.05; ** *p*<0.001.

**Table 8:** Linear regression analysis during Asian games.

	Unstandardized coefficients		Standardized coefficients	*t*	*p*	Collinearity diagnostics	
	*B*	*SE*	*Beta*			VIF	Tolerance
Constant	0.605	1.457	−	0.415	0.684	−	−
Gender	0.192	0.423	0.082	0.454	0.656	1.260	0.794
Age	−0.140	0.252	−0.094	-0.555	0.587	1.123	0.891
Training Years	0.758	0.280	0.517	2.708	0.016*	1.425	0.702
Trait anxiety	0.109	0.024	0.789	4.609	<0.001**	1.147	0.872
*R* ^2^	0.616						
*Adjust R* ^2^	0.514						
*F*	*F* (4,15) = 6.019, *p* = 0.004						
D-W	2.027						

Dependent variable: somatic anxiety.

VIF is designed to determine collinearity problems, and a value less than 5 indicates no collinearity. Tolerance = 1/VIF and the value less than 0.2 indicates collinearity; * *p*<0.05; ** *p*<0.001.

External training load was assessed by the total number of kilometers of the sessions. Internal training load was assessed by multiplying the athlete’s “rating of perceived exertion” (RPE, on a 1–10 scale) obtained 30 min after the completion of the training session by the duration (in minutes) of the session (Wallace et al., 2009).

Table 9 showed significant positive correlations between external and internal loads and the cognitive and somatic anxiety, and negative correlations with confidence during high volume training. This suggests that the level of competitive anxiety increases as external and internal loads increase.

**Table 9:** The correlation between training load and competitive anxiety at high training volume.

	Cognitive anxiety	Somatic anxiety	Confidence	External load	Internal load
Cognitive anxiety	1				
Somatic anxiety	0.494*	1			
Confidence	−0.626**	−0.508*	1		
External load	0.831**	0.724**	−0.701**	1	
Internal load	0.743**	0.757**	−0.629**	0.824**	1

* *p*<0.05; ** *p*<0.001.

## 4 Discussion

According to this study, there is no significant difference in trait anxiety and competitive state anxiety between male and female swimmers. This finding is consistent with the results of Guillén and Álvarez-Malé (2010). The reason for this lack of variability may be that the sample comprises the top elite swimmers in the country. These athletes have honed their skills through extensive competitive experience, leading to a leveling of anxiety levels between genders.

*Interviewee A3 said, “Everyone will anxiety in a competition, they just show it to a different degree or anxiety about different things, as well as different means of regulating it.” A4 expressed her opinion based on gender differences, “Who says women are not as good as men? Gender may lead to personality differences, but at this (top) level, girls perform just like men, hahaha.”*

This is an ongoing debate in the academic community about whether gender differences in competitive anxiety exist. While most studies indicate that female athletes generally have higher levels of competitive anxiety than male athletes (Amaro and Brandão, 2023), results can vary depending on the sport and population studied. Statistics show that since the Olympic Games, 80% of the medals won by the China’s swimming delegation have come from women’s events. China’s women’s swimming team has consistently excelled on the world stage often leading in competitiveness (Tan and Liu, 2003). since there is evidence in the literature that young swimmers of both sexes have similar characteristics of anxiety (Silva et al., 2017).

Yerkes’ inverted U-curve theory suggests that athletes perform best when their competitive anxiety is kept at an optimal level (Lee et al., 2020). This controlled anxiety can positively stimulate the nervous system, enhancing physical performance. In facing high-level swimmers worldwide, some athletes self-regulate to maintain their competitive anxiety at this moderate level, achieving better results (Alavizadeh et al., 2023). This study found that swimmers with strong performances have higher cognitive anxiety, but lower somatic anxiety compared to others. Swimmer A5, who broke his personal best in the World Championships, said, *“I was especially excited to be at the World Championships and watch so many masters. If you ask me if I was nervous or anxious, the answer is yes, but I seized at the chance to prove myself, and I felt as if I had been helped by God during the competition.”* These findings corroborate previous research, which showed that athletes participating in national and international competitions had lower SA scores, leading to an increase in performance (Souza et al., 2012; Silva et al., 2019).

This study shows significant differences in athletes’ competitive anxiety at different times. During the World Championships, swimmers experienced the highest levels of competitive anxiety, followed by the Hangzhou Asian Games and then the high training volume sessions. Psychology believes that anxiety is realised through cognitive evaluation, where athletes compare their technical, tactical, and psychological strengths with their opponents. When facing a particularly strong opponent, athletes may feel they have little chance of winning, leading to high psychological pressure and competitive anxiety (Zhang, 2001). Some scholars noted that in different levels of competitions, factors such as the strength of their opponents, Athletes’ personality traits and their internal psychological activities translate into external behaviours (Martens et al., 1990), resulting in significant differences in anxiety levels (Alavizadeh et al., 2022). The World Championships represent the pinnacle of swimming events, with the number of medals won serving as an indicator of a country or region’s competitive level (Sun and Zhao, 2023). The Fukuoka World Championships, being a major event before the 2024 Paris Olympics, attracts significant attention from various countries, reflecting their preparation for the upcoming Olympics. The results of related studies show that athletes with more training years have a higher cognitive anxiety, because they are afraid of losing (Silva et al., 2019).

According to the interview results, *swimmer A1 who has 19 years of training said, “The Fukuoka World Championships is the last world-class event before the Paris Olympics, I have to go all out to test my preparation and see how I compare to the world’s top athletes. I am not too worried about the Asian Games, I am aiming for a gold medal, but rather to make an impact on MVP. “*So, most athletes exhibited a high heart rate, sweating, and a heightened nervous system activity before the Fukuoka World Championships.

The Hangzhou Asian Games marks the third time China has hosted the largest international multi-sport event in Asia (Zhang and Cai, 2023). As a major event held “at home,” it has gathered significant expectations from various sectors. However, given the dramatic improvement in the level of Chinese swimmers and their obvious competition advantage in Asia, athletes’ internal motivation and pre-competition self-confidence were much stronger. By boosting self-confidence, athletes can optimise their performance (Kang and Jang, 2018). Consequently, better sports performance at the Asian Games correlated with lower levels of competitive anxiety, with more athletes reaching Level 1 than at the World Championships. This aligns with Terry’s findings, which suggest that athletes exhibit lower anxiety, higher self-confidence, and more positive attitudes and states when competing in home games (Terry and Parsons-Smith, 2021).

A2 commented, *“The Asian Games is a home event, and I cannot afford to be embarrassed. I am not too anxious about the level of my opponents. My friends and family will be there to cheer me on, so I’m determined to win this tournament to satisfy those who support me.”*

The present study demonstrated that high training volumes increased competitive anxiety in swimmers, which is consistent with previous findings (Chortane et al., 2022). Specifically, high training volumes can push athletes into an overtrained state, resulting in high RPE values, which elevate psychological fatigue and increase both cognitive and somatic anxiety (Collette et al., 2018). Typically, the body’s sympathetic and parasympathetic nerves balance each other, but during high training volumes, athletes experience heightened sympathetic activity, increased heart rate, and blood pressure. They also tend to hold their breath, resulting in low oxygen levels and increased muscular and mental tension (Williams et al., 2008). Scholars generally agree that athletes’ emotions are related to training load (Meeusen et al., 2013), this study suggests the increase in somatic and cognitive anxiety may lead to a decline in athletes’ self-confidence.

Athlete A6 remarked, *“These intense training sessions are really exhausting and frustrating, but they are necessary to win and improve. If I skip them, I cannot swim well, and that makes me anxious. It’s a vicious cycle.”* Studies have shown that muscle fatigue from high training volumes can prevent athletes from completing their training load, leading to a decline in well-being and self-confidence (Morgan and Hayward, 1988). According to researches, as training volume increases, athletes’ unhappiness rises, causing changes in thoughts, actions, and physiological responses. This decline in coping capacity affects their ability to meet goals, resulting in significantly decreased self-confidence (Chortane et al., 2022).

The present study demonstrated that, regardless of the moment of training and competition, state anxiety and trait anxiety in our elite swimmers were significantly positively correlated. This means athletes with high levels of trait anxiety also exhibited high levels of state anxiety, supporting Scanlan and Lewthwaite's (1986) findings. Someone’s results further showed that athletes with high trait anxiety experienced a greater increase in state anxiety during periods of heightened training volume (O'Connor et al., 1989). The interaction theory of anxiety, attentional control theory, and processing efficacy theory all agree that state anxiety results from a multidirectional interaction between an individual and their environment. These theories suggest that in stressful environments, such as competitions, athletes with high trait anxiety display higher state anxiety compared to those with low trait anxiety (Morris et al., 1981).

This is clearly illustrated by athlete A6: *“I often feel that I am not suited for competitive sports. I usually get anxious over small things, so during major competitions, I feel like I’m going to explode, but look at xx, who is always carefree, never overthinking, he can even fall asleep before an event, I really admire that level of relaxation.”*

Trait anxiety in athletes is persistent and varies individually as a personality trait. In contrast, competitive anxiety is a psychological state that occurs in specific contexts. These two forms of anxiety are related; high levels of trait anxiety can trigger more intense state anxiety in response to particular situations and psychological stimuli.

Translating research results into practical implications involves tailoring targeted interventions for individual elite swimmers. By identifying anxiety levels during different training and competition phases, sports psychologists, practitioners and safeguarding teams can take timely preventive measures to enhance effectiveness. Additionally, the environment surrounding elite swimmers must be ready to provide social support, especially during periods of increased pressure. Coaches should strengthen their psychological knowledge, understand the changing patterns of swimmers’ personality traits and anxiety states, and guide athletes in coping with competitive anxiety. It is also recommended to incorporate stage-by-stage simulated competitions into the training cycle to replace practice with competition, which can improve athletes’ motivation and self-confidence, and achieve desensitization (Chen et al., 2024).

One can note a few limitations to this research. The effect of other physiological-psychological variables (e.g., heart rate, blood lactate, heart rate variability) on competitive anxiety at various moments in the study remains unclear. Future studies could enhance the research by supplementing questionnaires with physiological-biochemical tests. Additionally, the number of subjects was limited because the study’s inclusion criteria required athletes selected for both the Fukuoka World Championships and the Hangzhou Asian Games in a single event. Future research could broaden the participant pool by selecting from the Olympic delegation. This study quantified results using World Aquatics Points. However, despite investigating changes in swimmers’ competitive anxiety at different points in time, the study was limited by its short duration. Further research is needed to examine changes in competitive anxiety levels at different stages of swimmers’ careers.

## 5 Conclusion

This study examined the characteristics of competitive anxiety impact sports performance across four different phases of competitive preparation, alongside its correlation with trait anxiety. Main results underline the competitive anxiety has different characteristics in the preparation cycle. It also provides support the trait anxiety is highly correlated with competitive anxiety. Responding to the findings of this study, professionals can develop personalised interventions aimed at managing swimmers’ competitive anxiety and enhancing their mental well-being, mitigate negative psychological impacts and boost self-confidence, thereby optimising their sports performance.

## Data availability statement

The raw data supporting the conclusions of this article will be made available by the authors, without undue reservation.

## Ethics statement

The studies involving humans were approved by the Sports Science Experiment Ethics Committee, Beijing sport University. The studies were conducted in accordance with the local legislation and institutional requirements. The participants provided their written informed consent to participate in this study.

## Author contributions

YZ: Conceptualization, Data curation, Formal analysis, Investigation, Software, Writing – original draft, Writing – review & editing. ZJ: Software, Visualization, Writing – review & editing. YW: Resources, Supervision, Writing – review & editing.
